# MLP-Based Regression Prediction Model For Compound Bioactivity

**DOI:** 10.3389/fbioe.2022.946329

**Published:** 2022-07-13

**Authors:** Yongfei Qin, Chao Li, Xia Shi, Weigang Wang

**Affiliations:** ^1^ School of Statistics and Mathematics, Zhejiang Gongshang University, Hangzhou, China; ^2^ Collaborative Innovation Center of Statistical Data Engineering, Technology and Application, Zhejiang Gongshang University, Hangzhou, China

**Keywords:** breast cancer drug candidates, biological activity, LASSO regression, MLP, neural

## Abstract

The development of breast cancer is closely linked to the estrogen receptor ERα, which is also considered to be an important target for the treatment of breast cancer. Therefore, compounds that can antagonize ERα activity may be drug candidates for the treatment of breast cancer. In drug development, to save manpower and resources, potential active compounds are often screened by establishing compound activity prediction model. For the 1974 compounds collected, the top 20 molecular descriptors that significantly affected the biological activity were screened using LASSO regression models combined with 10-fold cross-validation method. Further, a regression prediction model based on the MLP fully connected neural network was constructed to predict the bioactivity values of 50 new compounds. To measure the validity of the model, the model loss term was specified as the mean squared error (MSE). The results showed that the MLP-based regression prediction model had a loss value of 0.0146 on the validation set. This model is therefore well trained and the prediction strategy used is valid. The methods developed by this paper may provide a reference for the development of anti-breast cancer drugs.

## 1 Introduction

Cancer is an important global public health problem. Breast cancer is a malignant tumor that occurs in breast epithelial tissue. Breast cancer is one of the most common and deadly cancers in the world. Through the research of Batyrova et al. (2021). (2021), with the progress of society, economic development and environmental changes, the disease burden of breast cancer is gradually increasing. In 2021, global cancer data showed that the number of new cases of breast cancer in the world was as high as 2.26 million, surpassing the 2.2 million cases of lung cancer, which replaced lung cancer as the world’s leading cancer. Breast cancer causes serious damage to women’s life and health. 1 in 12 women will get breast cancer. Breast cancer is one of the most common malignant tumors in women, accounting for 7%–10% of female systemic malignant tumors. In recent years, breast cancer ranks first in the number of new cancer cases and deaths among women worldwide, enough to see that breast cancer has posed a serious threat to the health of women around the world. Although global medical care has improved since the early years, the pattern of breast cancer is not promising. As a chronic disease, it is not only a serious threat to the life and health of women all over the world, but also brings a burden in all aspects. Therefore, improving the cure rate of breast cancer is of great significance to women’s health.

With the rapid development of modern medical imaging technology and the deepening of biological research on breast cancer, the rate of early diagnosis of breast cancer has been increasing, which is of great significance for the early detection and timely treatment of breast cancer, as well as improving the five-year survival rate of patients. With the research on the biology of breast cancer, in addition to the early diagnosis of breast cancer, improving the cure rate of breast cancer and selecting more effective drugs for treatment are of great significance to medical research. Chemotherapy has been used as one of the main tools in the treatment of cancer (2021) (Cheng et al., 2021). There are many drugs available for the treatment, but most of these drugs are quite toxic, which may shorten the life span of cancer patients. Therefore, there is an urgent need to predict new compounds with minimal toxicity and strong biological activity through QSAR and ligand-based design (2016, 2022) (Nandi and Bagchi, 2016; Nandi et al., 2022). Today, with the dramatic increase in the number of drugs, in order to save time and cost, the most economical and reasonable way to research is to use the computer-aided artificial intelligence algorithm to predict and analyze the biological activity of drugs through the establishment of prediction models. Drug activity prediction is performed by collecting data on a range of compounds that act on breast cancer-related targets and their biological activity. The specific approach is that, for a disease-related target, collect a series of compounds acting on the target and their bioactivity data. Then, with a series of molecular structure descriptors as independent variables and the bioactivity value of compounds as dependent variables, a prediction model is constructed to predict and guide the structural optimization of existing active compounds. Zhao et al. (2021). (2021) have shown that FBXO15 plays a critical inhibitory function in regulating breast cancer progression. Some studies have found that Estrogen Receptor Alpha (ERα) is expressed in no more than 10% of normal mammary epithelial cells, but about 50%–80% of mammary tumor cells. In mice with ERα deletion, it was found that ERα plays an important role in breast development. Currently, anti-hormone therapy is commonly used in breast cancer patients with ERα expression, which regulates estrogen receptor activity to control estrogen levels in the body. Therefore, ERα is considered an important target for the treatment of breast cancer, and compounds that can antagonize ERα activity may be candidates for the treatment of breast cancer. For example, tamoxifen and raloxifene, the classic drugs for clinical treatment of breast cancer, are ERα antagonists whereas BSC-pyrazole and MPP can fully antagonize E2 stimulation of pS2 mRNA in MCF-7 breast cancer cells.

Based on the above, in this paper, an anti-breast cancer drug candidate screening model was established. We obtained bioactivity data of 1974 compounds targeting ERα for breast cancer treatment. Next, we downscale the data. Based on the data collected, a variable screening was first performed and 20 molecular descriptors with significant effects on biological activity were selected from more than seven hundred molecular descriptors. Finally, a regression prediction model was established based on MLP fully connected neural network to predict the bioactivity values of 50 new compounds. The main contributions of this paper are as follows:

• Several commonly used dimensionality reduction algorithms are compared in high-dimensional biomedical data screening, and the LASSO algorithm is selected to reduce the dimensionality of high-dimensional biomedical data, which reduces 729 dimensions to 20 dimensions and retains more information of the original variables, And the selected 20 molecular description variables cover most description types, accounting for a suitable proportion and representative. It greatly reduces the workload in the exploration of drug molecules and thus improves the efficiency of the preparation of new drug compounds.
• A multilayer perceptron (MLP) prediction model was used to predict the biological activity of the compounds, and the model prediction results are closer to the real values than other methods. The loss value of the REGRESSION prediction model based on MLP was 0.0146 in the validation set, indicating that the model had a good prediction effect. This is of great significance for the development of anti-breast cancer drugs.

## 2 Related Work

### 2.1 High-Dimensional Biomedical Data Variable Screening Methods

In recent years, with the development of biomedical testing technology, the amount of biomedical data accumulated in the research process has increased exponentially. In conventional statistical analysis, when conducting multivariate analysis, a certain ratio of the number of independent variables to the sample size is often required. However, the cumulative amount of biomedical data has resulted in a much larger number of independent variables than required, making it difficult to establish the correct association between the dependent and independent variables. In response to these problems, filtering out more critical variables from high-dimensional data has become an urgent problem for researchers.

Tibshirani et al., 1996 (1996) proposed the LASSO (Least Absolute Shrinkage and Selection Operator) estimate inspired by the penalty function. This method can compress certain constituents to zero within a reasonable choice of penalty parameters to achieve variable selection and perform parameter estimation. Then new methods of variable selection and contraction based on LASSO were gradually proposed, and other scholars also started to study and improve the LASSO method in depth. Fan and Li, 2001. (2001) proposed a penalized likelihood method called SCAD and proposed an optimization algorithm for this penalized likelihood function to address the problem that stepwise regression can be computationally costly and neglects the random errors that arise during model selection. Jolliffe et al. (2003). (2003) proposed an improved principal component method based on LASSO. Fonti and Belitser, 2017. (2017) explained and discussed how to use LASSO for feature selection. Efron et al. (2004). proposed a minimum angle regression algorithm to implement LASSO. Since the computational effort of this algorithm is the same as that of the least squares method, this has led to the widespread use of the LASSO regression method. Since the LASSO method is inconsistent with the variable selection of some cases, Zou et al., 2006 (2006) proposed the adaptive LASSO method to overcome this problem of LASSO. Adaptive LASSO has Oracle properties and its performance remains constant under certain canonical conditions when extending it to generalized linear models. Zou et al. (2006). (2006) used LASSO to give modified principal components with sparse loadings for enhancing the interpretability of the principal components. For some strongly correlated data, such as medical data, biological data, etc., LASSO and Adaptive LASSO cannot select all highly correlated variables into the model.

Zou and Hastie, 2005. (2005) proposed the Elastic Net method by improving the LASSO method, which is a new regularization and variable selection method that can handle the complex collinearity problems in covariate. Zou and Zhang, 2009 (2009) further proposed the Adaptive Elastic-Net (AEN) method, which weights each regression coefficient of the primary penalty term so that it has the properties of both the adaptive LASSO and Elastic Net methods. Li et al., 2017. (2017) applied the Elastic Net approach to variable selection in a balanced longitudinal data model and demonstrated the compatibility and group effect properties of the approach. Lin and Yang, 2019. (2019) extended non-negative adaptive Elastic Net estimation to a high-dimensional sparse linear model and proved its Oracle property and validity under some canonical conditions with valid samples. Existing studies have demonstrated the good nature of LASSO and Elastic Net for variable screening. Yamada et al. (2014). (2014) verified the effectiveness of LASSO method through the feature selection experiment of classification and regression of thousands of features. Muthukrishnan and Rohini, 2016. (2016) explored the features of the popular regression methods, OLS regression, ridge regression and the LASSO regression. Zhang et al. (2021). (2021) used LASSO dimensionality reduction method to conduct experiments on the combination of feature sub models to obtain the best top-level feature number, thus providing support for the effective prediction of DNA binding proteins. In this paper, the original 729 variables were screened using the LASSO method according to the research objectives.

### 2.2 Biological Activity Prediction Model

Machine learning algorithms are widely used in biological activity prediction. Scholar Jia et al., 2019 (2019) used the RF algorithm combined with ten-fold cross-validation to build a quantitative prediction model for drug targets. The MSE of the RF model constructed on the EC50 validation set and test set were both less than 0.09, and the R2 was greater than 0.96; in the KD data set, the MSE is less than 0.12, and the R2 is greater than 0.94. Lv and Xue, 2011. (2011) tested the classification prediction model of SVM in hepatitis virus NS5B protease inhibitor and non-inhibitor, which had high model calculation efficiency and prediction accuracy. He and Zhu, 2020. (2020) modeled the drug target prediction problem with reference to the recommendation system, and made improvements to the traditional algorithm, by adding drugs and targets double regularization to improve the accuracy of the model. Based on different application scenarios, the advantages of various algorithms are different. The K-Nearest Neighbor algorithm performs well in the classification of known Hepatitis C Virus NS5B protease inhibitors and non-inhibitors. Random forest is suitable for quantitative prediction of drug EC50 and CD value. Liu et al. (2013). (2013) proved Support Vector Machine is good at processing the prediction of drug-target BuChE inhibitors and non-inhibitors. Collaborative filtering can solve the problem of sparse matrix by the mixing weighted drug-target association matrix, so it is suitable for the recommendation of “drug-target” and the prediction of “drug-target” interaction (2017) (Zhang, 2017). The artificial neural network is suitable for predicting the inhibitory activity of drug molecules against p38R MAPase (2019) (Abdolmaleki and Ghasemi, 2019).

The neural network model originated from the mathematical model of MP neurons proposed by McCulloch and Pitts in 1943. MLP model is a popular and practical one among many neural network models. Pinkus et al., 1999. (1999) discussed various approximate theoretical problems in the MLP model of neural networks. Then, Rosenblatt et al., 1958 (2017) proposed a single-layer perceptron model, but it could only distinguish basic shapes such as triangles and squares. In 1986, the second-generation neural network was proposed (2016) (Jiao et al., 2016), which introduced the Sigmoid function as the activation function, and used multiple hidden layers instead of the original single fixed feature layer to solve the nonlinear classification problem. The back-propagation algorithm was used to train the model, and the back-propagation algorithm and its improvement still play an important role in model training so far. Multilayer perceptron (MLP), also known as the feedforward neural networks, has been widely used in research. Gao et al. (2019). (2019) constructed a multilayer neural network model to accurately assess the risk of non-contact sports injuries in rugby. Xiao et al. (2021). (2021) constructed and verified the prediction model of occupational coal worker’s pneumoconiosis (CWP) incidence based on multilayer perceptron neural network, and discussed its application value in the prediction of CWP incidence. Generally speaking, MLP falls into two categories: supervised and unsupervised (2021) (Dua et al., 2021). The trainer is responsible for training the MLP. It has the highest performance for new input datasets. The research in this paper is to predict the activity of new compound molecules with better biological activity, so it is suitable to use artificial neural network for prediction. In order to further optimize the effect of improving prediction, MLP neural network is applied.

## 3 Methods

### 3.1 The LASSO Algorithm

LASSO (Least Absolute Shrinkage and Selection Operator) is a variable selection method proposed by the statistician Tibshirani. The basic idea of which is to introduce a penalty factor to the ordinary least squares estimation to penalise or constrain the estimator β by the $L_{1}$ norm. For a data set with n predictor-response variable pairs ${\left\{\left(x_{i},y_{i}\right)\right\}}_{\left(i=1\right)}^{n}$ , LASSO seeks an estimate $\hat{\beta }$ that fits the data better by minimizing the RSS $\left(\hat{\beta }\right)$ .

$$RSS\left(\hat{\beta }\right)=\underset{\hat{\beta }}{\underset{︸}{\arg\min}}{\sum_{i=1}^{n}\left(y_{i}-\sum_{j=1}^{p}\beta_{j}x_{ij}\right)}^{2}+\lambda \sum_{j=1}^{p}|\beta_{j}|$$ (1)

Where λ≥0 is a penalized parameter, $\lambda \sum_{j=1}^{p}\left|\beta_{j}\right|$ is a compression penalty.

$$\hat{\beta }=\arg\min\left\{{\left\|y-x\beta \right\|}_{2}^{2}\right\}\;\;\;\;\;\;\;\;\;\;\;\;\;\;s.t.\left\|\beta \right\|\leq t$$ (2)

t≥0 is a turning parameter, which controls the intensity of compression. If the parameter obtained by ordinary least squares estimation is denoted as ${\hat{\beta }}^{0}$ , then LASSO achieves compression, as long as $t<\sum_{j=1}^{p}\left\|{\hat{\beta }}_{j}^{0}\right\|$ . And for some models with small absolute values, the coefficients can be compressed to zero. Thus, the inequality ‖β‖≤t can effectively constrain the parameter space and allow the final model to be well interpreted.

### 3.2 The Perceptron Model

The single-layer perceptron model was the first and simplest neural network model to be proposed.

**FIGURE 1:**
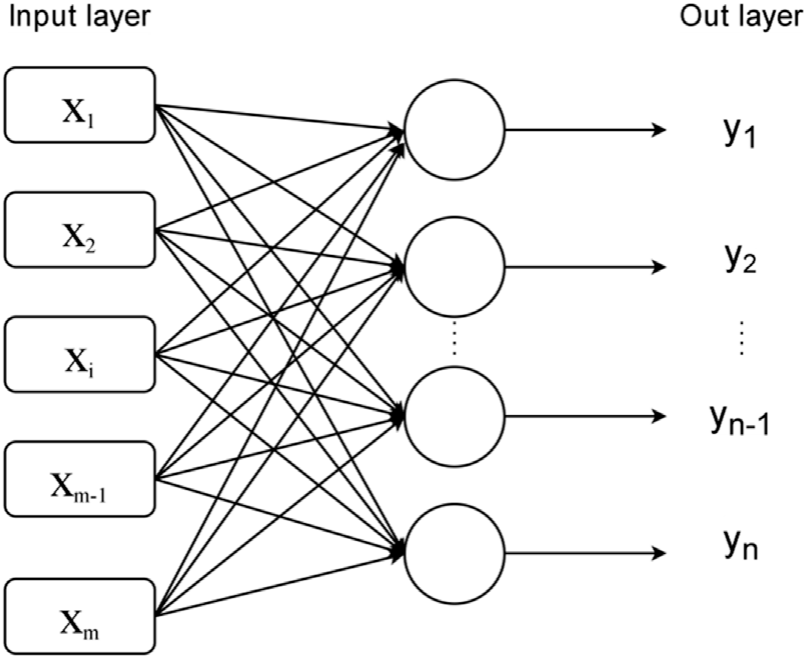
Perceptron model.

As shown in the Figure 1, it has two layers, the input layer and the output layer, which are each composed of multiple ANs and are interconnected.

In terms of the operation principle, the single-layer perceptron first receives input data $\left(x_{1},x_{2},...,x_{m}\right)$ at the input layer, and then accumulates the input data with their respective weights, That is, for the Kth output layer neuron, the total amount of information it receives is $\sum_{i=1}^{n}w_{ik}x_{i},k=1,2,...n$ . So the output of the Kth output layer neuron is:

$$y_{k}=f\left(\sum_{i=1}^{m}w_{ik}x_{i}-\theta_{k}\right)$$ (3)

The model of a single-layer perceptron actually constructs some hyperplanes to separate different data sets in a high-dimensional space. So it can handle linearly separable problems better, but it is helpless for linearly indistinguishable data sets. In order to further improve the learning ability of neural networks to solve more practical problems, the multilayer perceptron model, which evolved from the single-layer perceptron, was naturally created.

**FIGURE 2:**
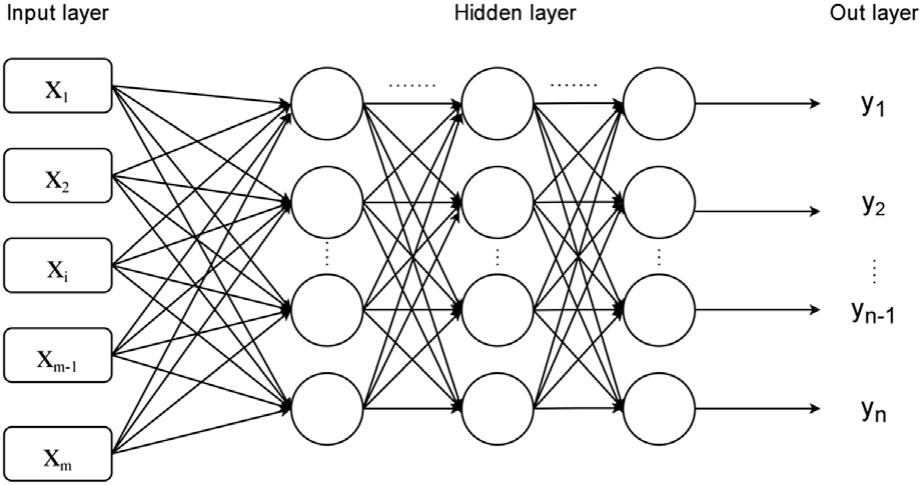
Multilayer perceptron model.

As shown in the Figure 2, in addition to the input layer and output layer, the multilayer perceptron model also includes a hidden layer, which is also composed of multiple layers of artificial neurons, and each layer is fully connected to each other. In 1991, Kurt Hornik proposed the Universal Approximation Theorem, which states that if the hidden layer can consist of any number of artificial neurons, then a feedforward neural network containing a hidden layer can approximate any continuous function in the real number range. This theorem actually tells us that a multilayer perceptron neural network model with hidden layers can solve more problems than a single layer perceptron model. Due to the large number of data indicators and high dimensions involved in this paper, in order to make the prediction model in this paper have higher prediction accuracy, the multilayer perceptron model is chosen to construct the prediction model.

## 4 Experiments and Results

### 4.1 Data Set and Data Preprocessing

For the breast cancer therapeutic target ERα, the required data were obtained from the University of Alberta’s DrugBank database of drug molecules, which is a unique bioinformatics and cheminformatics resource that combines detailed drug data with comprehensive drug target information for the study of drug mechanisms and even the exploration of novel drugs. The data used in this paper contains a total of 1974 compounds and the corresponding bioactivity values for ERα, IC_50,_ and pIC_50_. And the properties of each compound are co-represented by 729 molecular descriptors, which will also be added as independent variables in the Quantitative Structure-Activity Relationship (QSAR) model. In particular, pIC_50_ is the negative logarithm of the IC50 value, which usually has a positive correlation with biological activity, i.e., a higher pIC_50_ value indicates higher biological activity. The pIC_50_ is generally used to represent biological activity values in practical QSAR modelling.

As the data collected for the molecular descriptors are two-dimensional, i.e., information corresponding to the solubility and surface area of the molecule. Therefore, certain characteristics that have the greatest impact on the results need to be filtered out as feature data when building the model. Based on the data collected, the importance of bioactivity values of compounds was ranked according to molecular descriptors (independent variables) to achieve the purpose of variable selection. This means that we need to find a metric to compare the importance of the variables cross-sectionally.

Due to the large number of variables, in order to explore whether there is multicollinearity among 729 variables, this paper analyzed the correlation between molecular descriptor variables by solving the correlation coefficient matrix. After arriving at a judgement based on the correlation matrix, an appropriate regression model will be built to describe the importance of the variables, thus enabling the screening of the molecular descriptors.

The first step was data pre-processing, reading the molecular descriptor data as the independent variable and the pIC50 bioactivity value data of ERα as the dependent variable. And the results of data missing judgment indicated that there were no data set missing, and the data can be directly normalized by Z-score.

In the second step, the Pearson correlation coefficient ρ was used to calculate the correlation coefficients between the respective variables. And the correlations between the molecular descriptor variables will be analyzed using the correlation coefficient matrix.

$$\rho_{XY}=\frac{COV\left(X,Y\right)}{\sqrt{D\left(X\right)\sqrt{D\left(Y\right)}}}=\frac{E\left(\left(X-EX\right)\left(Y-EY\right)\right)}{\sqrt{D\left(X\right)\sqrt{D\left(Y\right)}}}$$ (4)

Where Cov(X,Y) is the covariance of *X* and *Y*, and D(X) , D(Y) denote the variance of *X*, *Y* respectively. The correlation coefficients between 729 variables can be calculated according to this formula.

**FIGURE 3:**
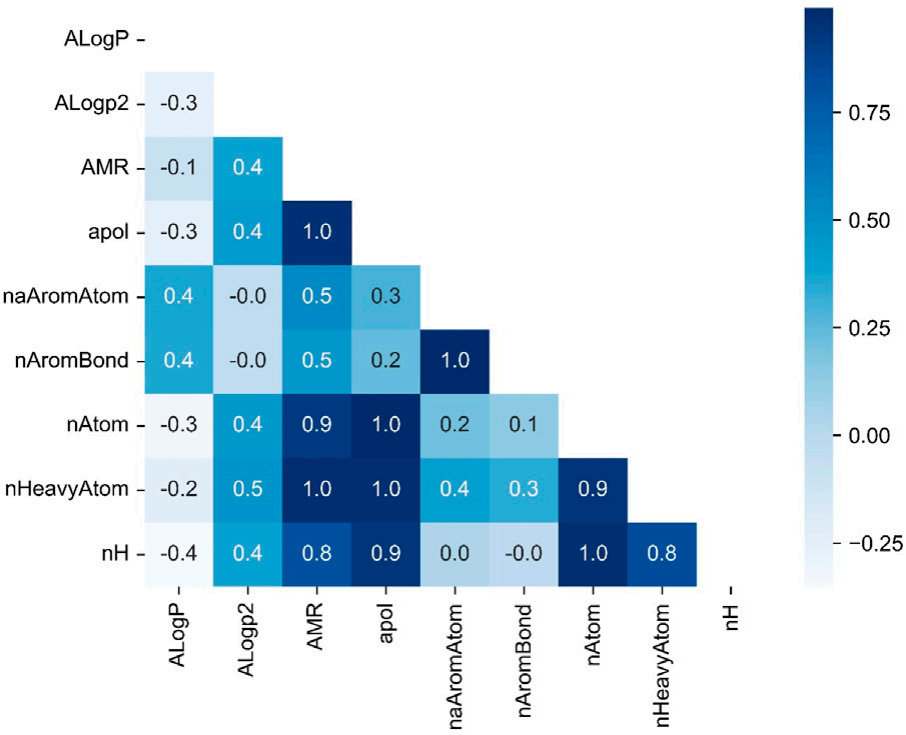
Diagonal matrix plot of correlation coefficients (10 variables).

The diagonal matrix of the correlation coefficients between 10 arbitrarily selected variables is shown in the Figure 3. From the figure above, it can be obtained that there is a close correlation between some of the molecular descriptor variables. For example, the correlation coefficients between nAtom and AMR, apol reached 0.9 and 1.0 respectively, indicating the existence of multicollinearity when using this data set for regression analysis.

### 4.2 Filtering Molecular Descriptors Based on the LASSO Algorithm

#### 4.2.1 LASSO + 10-Fold Cross-Validation

**FIGURE 4:**
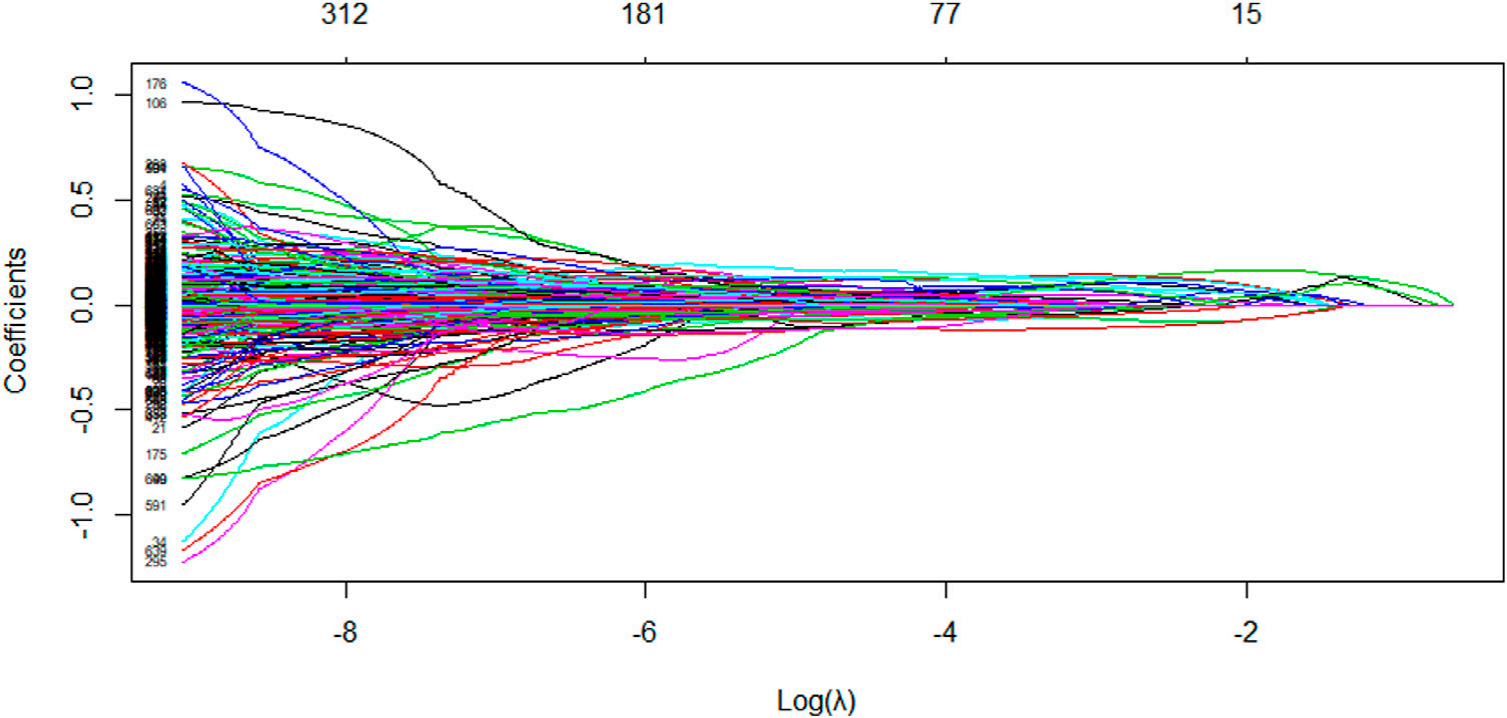
Plot of the variation of the L1 parity against the regression coefficient.

The LASSO algorithm is used to obtain a more refined model by constructing a penalty function that compresses the coefficients of less relevant variables to zero and removes these characteristic variables. The LASSO algorithm performs variable screening while fitting a generalized linear model. This can effectively avoid the over-fitting problem caused by too many variables and also help to solve the multicollinearity problem in the correlation analysis results of the previous step. Based on the LASSO algorithm, the molecular descriptor data were entered into the R software and the number of calculations was set to 1,000. The model stopped at 919 runs and the algorithm converged to the optimal solution, which contained 345 non-zero coefficients, i.e., 384 variables were initially eliminated, corresponding to a regularization parameter λ value of 0.00011. The Figure 4 shows the evolution of the model coefficients.

This paper continued to use k-fold cross-validation method in conjunction with the LASSO regression algorithm to increase the accuracy of the model fit. In the cross-validation stage of the model, the 10-fold cross-validation was performed by setting random number seeds. The mean squared error (MSE) was used as the target parameter to minimize the loss, so that the regularization parameter λ that minimize the loss could be filtered.

**FIGURE 5:**
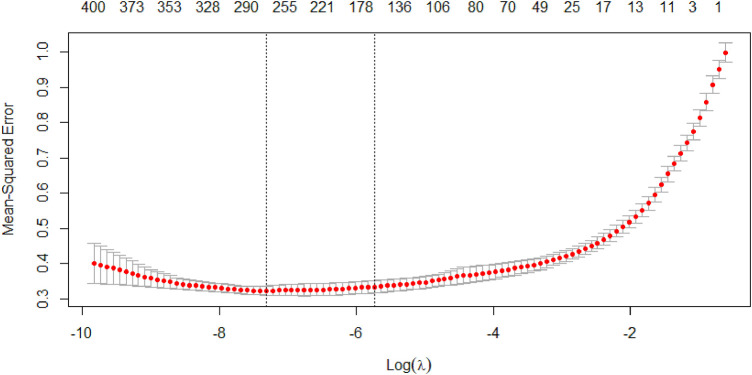
MSE with Log(λ) curve.

The vertical coordinate of the Figure 5 shows the mean squared error of the model, which changes as the penalty increases. And the horizontal coordinate at the top of the graph shows the number of independent variables. The first dashed line shows the logarithm of λ when the mean squared error is smallest, and the second dashed line shows the logarithm of λ when it is one standard error away from the minimum mean squared error. The two values are printed in the Table 1.

**TABLE 1:** Cross-validating lambda values.

Measure: Mean Squared error					
Lambda		Index	Measure	SE	Nonzero
Min	0.000663	73	0.3232	0.01272	281
1se	0.003225	56	0.3344	0.01698	163

In general, the latter is chosen as the optimal value of λ . The model given by 1se has a better performance and has fewer independent variables. Since the objective of this section is to filter the independent variables, the 1se value is also chosen as the optimal solution. Therefore, the optimal λ value of the 10-fold cross-validated LASSO regression model was 0.003225, and the corresponding regression coefficients of each variable under this value were further obtained.

A total of 163 variables were retained in the 10-fold cross-validated LASSO regression model, which further eliminated 182 variables compared to the non-cross-validated model. The absolute values of the regression coefficients of the optimal model were then taken to reflect the importance of the variables on the pIC_50_ bioactivity values. The Table 2 shows the top 20 molecular descriptors with the most significant effect on pIC_50_ bioactivity values.

**TABLE 2:** Molecular descriptors with significant effect on pIC_50_ bioactivity values (TOP20).

Variable	Coefficient	Abs_coeff
nHBAcc	−0.34921	0.349,211
SP-7	−0.25978	0.259,782
C3SP2	0.198,333	0.198,333
ndO	0.16484	0.16484
nsssCH	0.163,161	0.163,161
ECCEN	0.145,899	0.145,899
MLFER_A	0.140,239	0.140,239
mindO	−0.13936	0.139,355
ATSm5	0.138,853	0.138,853
minHAvin	0.137,933	0.137,933
MDEC-34	−0.12988	0.129,875
BCUTp-1h	0.129,423	0.129,423
SsF	0.123,461	0.123,461
mindsCH	−0.12217	0.122,166
C1SP3	0.117,622	0.117,622
maxHsOH	0.11567	0.11567
nHBint7	−0.11027	0.110,271
MDEN-22	0.099217	0.099217
ATSp5	−0.09359	0.093587
SHBint8	0.091518	0.091518

#### 4.2.2 Evaluation of Screening Results

This section evaluates the performance of the model by using two accuracy evaluation indicators, explainable deviation (%Dev) and mean squared error (MSE).

**FIGURE 6:**
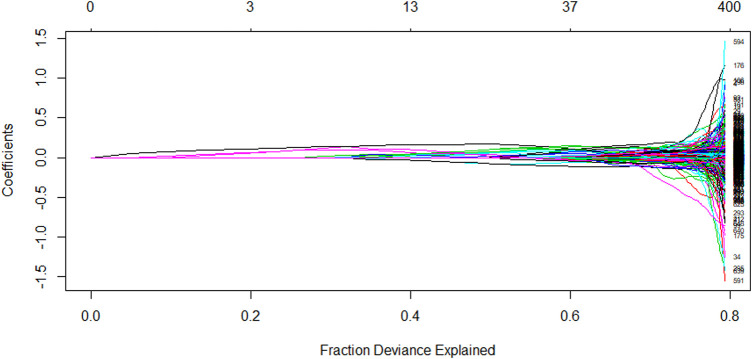
Plot of model explainable deviation against variable coefficients.

The Figure 6, with the number of independent variables indicated by the horizontal coordinate, shows the relationship between the explainable deviation and the variable coefficients for a 10-fold cross-validated LASSO regression model. The explainable deviation means the amount of sample information contained in the model, and the larger the value, the better. For example, with the number of independent variables exceeding 37, the model was able to explain 60% of the sample information. With the aforementioned λ value equal to 0.003225, the explainable deviation value of this model was 78.96, which indicates that the model has good model properties, and is able to explain 78.96% of the sample information. At the same time, the mean squared error of the model was very close to the minimum mean squared error, which can be concluded that the 10-fold cross-validated LASSO regression model constructed in this paper worked well and can achieve the purpose of screening the variables.

**FIGURE 7:**
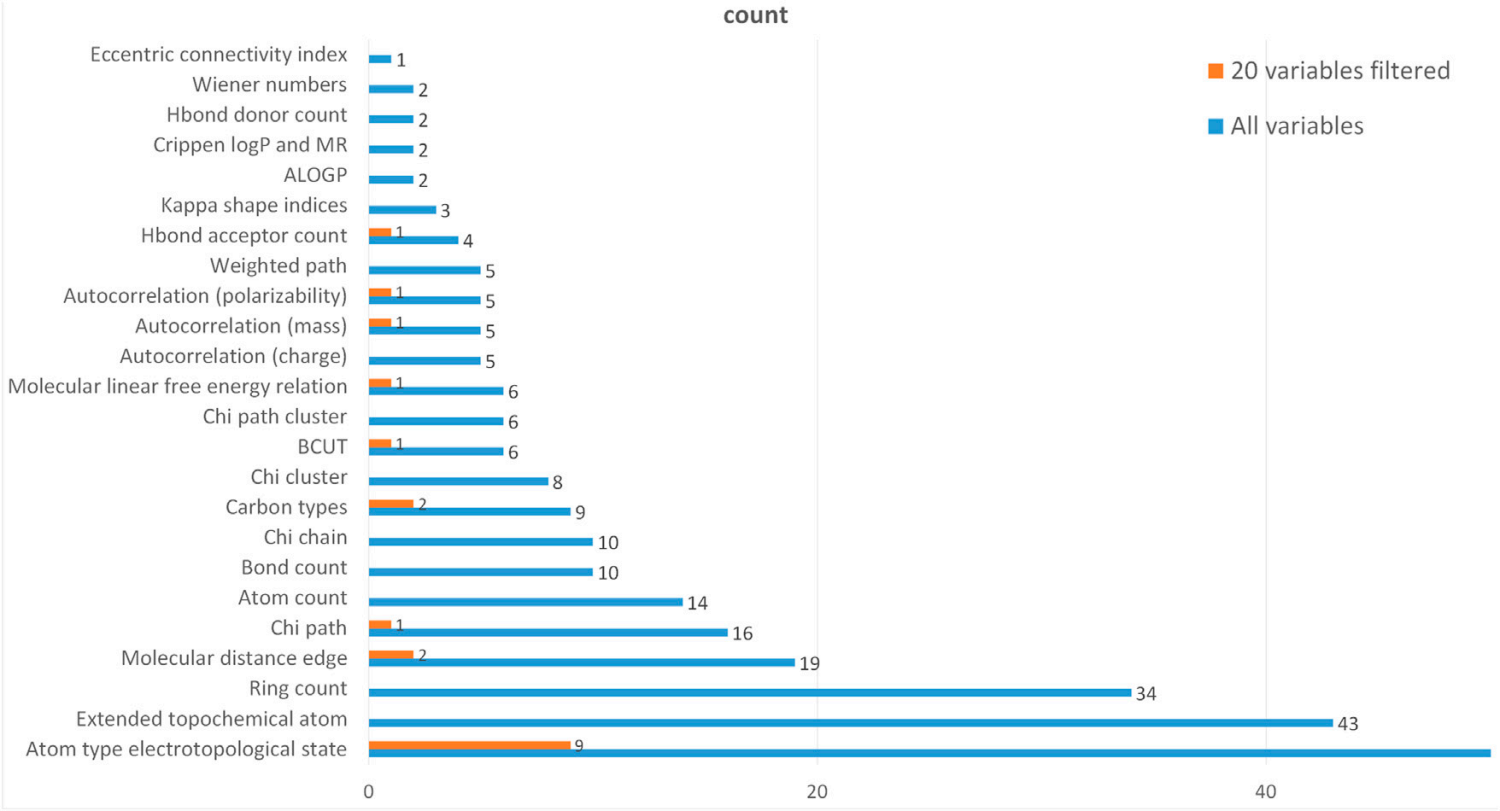
Filter variable classification statistics.

To further verify the rationality of the variables selected in the above model, the relationship between the corresponding type of the selected molecular descriptors and the corresponding type of all molecular descriptors is statistically shown in Figure 7.

In Figure 7, the blue one represents the categorical statistics of 729 variables and the orange one represents the categorical statistics of 20 variables screened by LASSO regression. Through searching, 46 types 727 variables were determined among 729 molecular descriptive variables. Because 50% of the descriptor types contain only one molecular descriptor, they are not shown in the graph. The category “Atom type electrotopological state” contains the largest number of descriptors, 488 in total. It is clear from the graph that the top 20 molecular descriptor variables screened by the LASSO regression cover most of the descriptor types and the proportion is appropriate, which can be considered that the variable screening is representative.

### 4.3 Quantitative Structure-Activity Relationship Model Prediction Analysis Based on MLP Neural Network

After dimensionality reduction of the molecular descriptor data, the top 20 molecular descriptor variables with significant influence on the bioactivity values were obtained. The information contained in these variables was then learned to find further relationships between compounds and ERα bioactivity values. In this section, the MLP neural network was trained to learn and the model relationships were used to predict the IC_50_ and pIC_50_ values of 50 new compounds.

#### 4.3.1 Development of an MLP Neural Network Prediction Model for Biological Activity Values

MLP generalises the perceptron to some extent and its weakness of lacking the ability to recognise linear indivisible data is well overcome by MLP neural networks. In machine learning, there is generalized linear model algorithm, which can be formulated as:

$$\hat{y}\left(w,x\right)=w_{0}+w_{1}x_{1}+\ldots +w_{p}x_{p}$$ (5)

The MLP could be considered as a multi-layer processing algorithm for the generalized linear model. The linear weighted combination of the above formula needs to be carried out on each layer, and the final desired predicted value y can be obtained after n layers. Therefore, the MLP can be understood as a multi-layer processing model for the generalized linear model, which can be used for classification and regression prediction processing in the machine domain. We selected its function of regression prediction to make regression prediction of pIC_50_ value of the new compounds.

**FIGURE 8:**
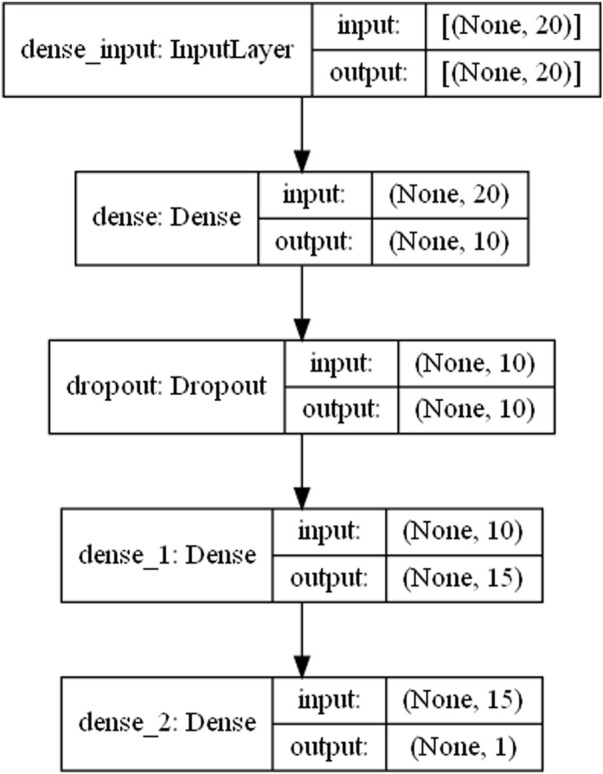
Network structure diagram.

The Figure 8 shows the network structure of the model constructed in this paper. The first layer is an input layer, the second layer is a fully connected layer with an excitation function of relu, and the third layer is a discard layer, which discards the neuron links with the fourth layer with a certain probability to prevent overfitting. In this model, the probability of discarding neuronal links was set to 0.2. The fourth layer is also a fully connected layer. The last layer is the output layer. And the linear excitation function was selected as the excitation function.

#### 4.3.2 Analysis of MLP Neural Network Results

Based on the MLP neural network model, the 1974 sample data were divided into a training set and a validation set, in the ratio of 7:3, and the model was trained with the training set data and then validated on the validation set. In the setting of the neural network model parameters, for the fitting of the model loss, the mean squared error (MSE) was used as the loss function value (loss) to measure the effectiveness. The number of epoch iterations was set to 200, and the size of the batch data used for gradient descent each time was also set to 200 for model training.

**FIGURE 9:**
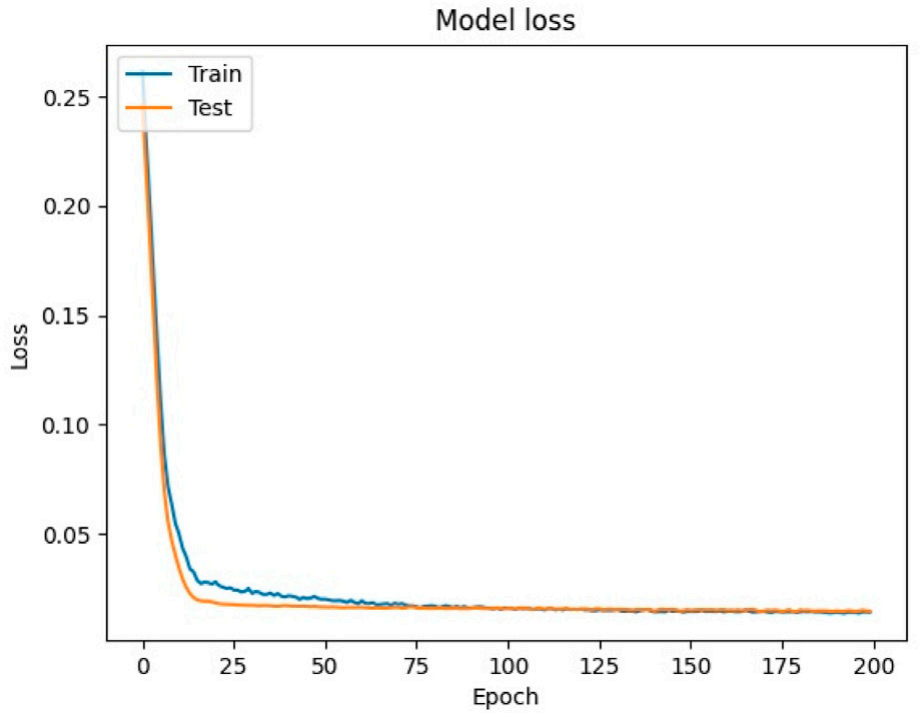
Loss function diagram.

By visualizing the training history, it can be seen in Figure 9 that the loss function values of the training and validation sets show a clear downward trend. The loss of both sets shows a sharp decreasing trend when the epoch was trained to around 10. Then, the loss of the training set shows a very small oscillation when the epoch was trained to between 20 and 50, while the validation set still keeps decreasing smoothly. The final loss value of the validation set was 0.0146, indicating that the model has a good training effect. At the same time, the loss function curve of the validation set is always below the loss function curve of the training set, indicating that the model did not show any overfitting phenomenon. So it can be considered that the model has good regression effect and can be used for the prediction of the test set.

Through the quantitative prediction model of ERα bioactivity constructed above, the bioactivity values of 50 new compounds were predicted. The results of the IC_50_ and pIC_50_ values are shown in Table 3, where the IC_50_ values were further solved from the negative logarithmic relationship between the two.

**TABLE 3:** Table of predicted results.

Index	IC50_nM	pIC50	Index	IC50_nM	pIC50
1	625.279,085	6.203,926	26	7,550.487,226	5.122,025
2	1,514.918,949	5.819,611	27	2,975.551,524	5.526,433
3	1,230.488,010	5.909,923	28	1,056.888,664	5.975,971
4	80.848,224	7.092329	29	154.518,702	6.811,019
5	48.221,256	7.316,762	30	242.322,471	6.615,606
6	39.612,235	7.402,171	31	3,283.679,462	5.483,639
7	2.341,306	8.630,542	32	8,099.091247	5.091564
8	23.148,631	7.635,475	33	1947.127,430	5.710,606
9	47.355,375	7.324,631	34	8,526.529,200	5.069228
10	34.096260	7.467,293	35	8,391.913,980	5.076139
11	31.653,379	7.499,580	36	472.583,992	6.325,521
12	32.697,205	7.485,489	37	466.080905	6.331,539
13	25.456,401	7.594,203	38	4,262.275,525	5.370,359
14	23.424,709	7.630,326	39	458.985,483	6.338,201
15	9.698,761	8.013284	40	441.836,676	6.354,738
16	9.306,946	8.031193	41	426.397,463	6.370,185
17	26.925,417	7.569,838	42	429.814,207	6.366,719
18	36.208,316	7.441,192	43	662.573,029	6.178,766
19	157.062175	6.803,928	44	145.994,719	6.835,663
20	1953.101,219	5.709,275	45	426.397,463	6.370,185
21	26.404,043	7.578,329	46	5,860.424,608	5.232,071
22	2,333.532,620	5.631,986	47	5,528.679,594	5.257,379
23	894.967,968	6.048193	48	5,671.119,197	5.246,331
24	1,616.214,377	5.791,501	49	4,657.397,312	5.331,857
25	8,979.197,446	5.046763	50	996.189,669	6.001658

## 5 Conclusion

To address the problem of compound screening and bioactivity prediction in the anti-breast cancer drug candidates development process, this paper used a computer-aided approach combined with a neural network algorithm to conduct the analysis. Based on the molecular descriptor information and bioactivity values of compounds, and etc., correlation analysis was used to gain a preliminary understanding of the linkage between the independent variables, and the results indicated the existence of multicollinearity between them. Therefore, the LASSO regression algorithm was used to assess the feature importance of 1974 compounds. The features were further screened in combination with cross-validation method, so as to determine the importance of molecular descriptors on biological activity. At the end, the top 20 molecular descriptors with the most significant impact on biological activity were screened. It can be seen from the variation curve of explainable deviation and mean squared error of the model, as well as the statistical graph of the type of variables, that the established variable screening model is effective and the results are well representative. The MLP neural network was then used for learning. The data were divided into training and validation sets to construct a quantitative prediction model of the compound molecular descriptor variables and the bioactivity value pIC_50_. The final validation set loss function value was 0.0146.

The results showed that the molecular descriptors screening model and bioactivity value prediction model were reasonable, and the applicability of the model was verified. The predictive analysis of the compound molecular descriptors could provide an effective reference for the development of anti-breast cancer drug candidates.

## Data Availability

The original contributions presented in the study are included in the article/Supplementary Material, further inquiries can be directed to the corresponding author.
